# Plasmonic Toroidal Dipolar Response under Radially Polarized Excitation

**DOI:** 10.1038/srep11793

**Published:** 2015-06-26

**Authors:** Yanjun Bao, Xing Zhu, Zheyu Fang

**Affiliations:** 1 School of Physics, State Key Lab for Mesoscopic Physics, Peking University, Beijing 100871, China; 2 Collaborative Innovation Center of Quantum Matter, Beijing 100871, China

## Abstract

Plasmonic toroidal resonance has attracted growing interests because of its low loss electromagnetic properties and potential high sensitive nanophotonic applications. However, the realization in a metamaterial requires three-dimensional complicated structural design so far. In this paper, we design a simple metal-dielectric-metal (MIM) sandwich nanostructure, which exhibits a strong toroidal dipolar resonance under radially polarized excitation. The toroidal dipole moment as the dominant contribution for the scattering is demonstrated by the mirror-image method and further analyzed by Lagrangian hybridization model. The proposed toroidal configuration also shows a highly tolerant for misalignment between the structure center and the incident light focus. Our study proves the way for the toroidal plasmonic application with the cylindrical vector beams.

Toroidal dipole moment, characterized by currents flowing on the surface of a torus along its meridians, was first considered by Zel’dovich^1^ to explain parity violation in the weak interaction in nuclear physics. The static toroidal moment has been found in many natural materials, including ferroelectric^2^, ferromagnetic^3^ and magnetoelectric structures^4^,^5^, as well as biological materials^6^,^7^. Since the toroidal dipole moment violates both the space-inversion and time-reversal symmetries, many intrigue phenomena such as non-reciprocal refraction of light^8^, lasing spaser^9^ and dichroism^10^ have been predicted. Although the importance of static toroidal moment in solid state systems and particle physics has been recognized, the dynamic toroidal dipole moment in the classical electromagnetism is less known. Unlike the conventional dynamic multipoles, the toroidal dipole moment is not included in the standard multipole expansion^11^. With the intensity much weaker than the electric and magnetic resonance, the toroidal response is extremely hard to be observed in the experiment.

Metamaterial, an artificial sub-wavelength structure, that is engineered to have properties not available in natural materials, has become a subject of growing interest owing to a number of appealing applications such as artificial magnetic response^12^, cloaking^13^, negative refraction^14^,^15^ and perfect absorber^16^. Recently, by properly arranging four three-dimensional split rings in a unit cell, Kaelberer *et al.* demonstrated the existence of the toroidal dipolar resonance in the microwave frequency^17^ and Huang *et al.* further pushed its resonance to the optical frequency by scaling down the split-ring size^18^. Subsequently, various structures, such as asymmetric double-bars^19^, multifold double-rings^20^, oligomer nanocavities^21^, and other structures^22^,^23^,^24^,^25^,^26^,^27^ were proposed to support the dominant toroidal resonance. However, because the electromagnetic field distribution of linearly polarized light preserves space-inversion symmetry, geometric asymmetries are always introduced for the aim of space-inversion symmetry breaking^17^,^18^,^19^. This makes the structure complicated, which is quite a challenge for the modern nanofabrication especially at optical frequencies.

Cylindrical vector beam (CVB, such as radially and azimuthally polarized light) is a special light source with highly symmetric electromagnetic field distribution and ultra-small focal spot size (0.07λ^2^)^28^,^29^. Due to their unique polarization properties, the CVB is an excellent light source for the investigations of light-matter interaction in nano-optics, like high resolution imaging^30^,^31^, plasmonic focusing^32^,^33^, and other nanoplasmonic applications^34^,^35^,^36^,^37^,^38^. In this paper, we aim to eliminate the aforementioned challenge of the toroidal metamaterial design. Under the radially polarized light with inherently broken space inversion symmetry, the toroidal dipolar moment can be strongly excited in a metal-dielectric-metal (MIM) sandwich nanostructure. In addition, mirror-image method and Lagrangian hybridization model are used to understand and analyze the finite difference time domain (FDTD) simulation results.

## Results

**Figure 1:**
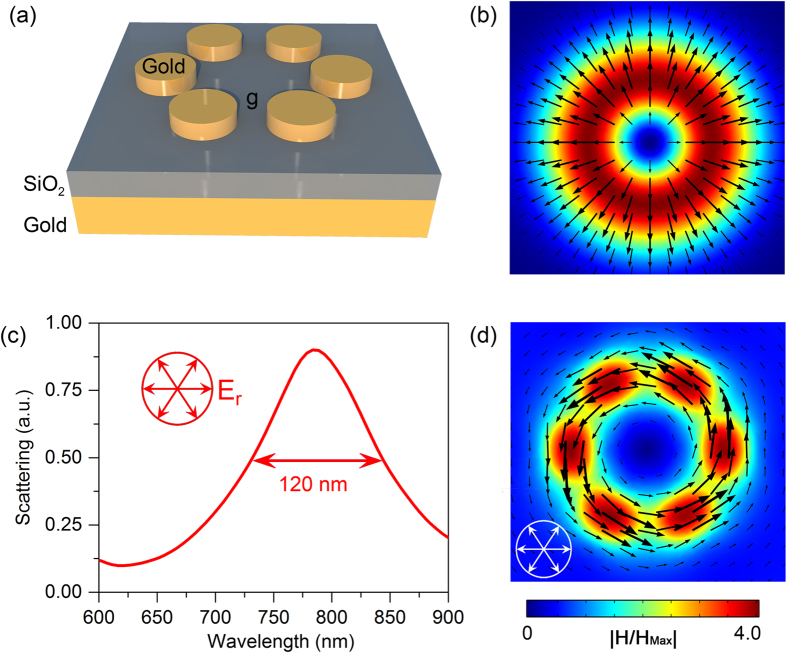
Structure of the toroidal structure and its optical response under radially polarized light. (**a**) A schematic view of the toroidal structure composed of a gold hexamer and metallic mirror separated by a dielectric layer. The diameter and thickness of the gold disks are 120 nm and 30 nm, respectively. The thickness of the SiO_2_ spacer is 30 nm and the thickness of the gold mirror is 60 nm. The gap size between two neighbor gold disks is *g* = 50 nm. (**b**) The intensity and field profiles of radially polarized light. The black arrows indicate the vectors of the incident electric fields. (**c**) The FDTD calculated scattering spectra of the toroidal structure under radially polarized light. (**d**) The simulated magnetic field at the center of the dielectric layer at the resonance peak of 785 nm. The black arrows represent the vectors of the magnetic fields.

Figure 1a illustrates the geometry of the proposed toroidal structure, which is composed of a gold hexamer and a bottom gold mirror separated by a layer of silicon dioxide (SiO_2_). The numerical simulations were performed by using FDTD method. Radially polarized light with electric field parallel to the radius vector from the axis (Fig. 1b) was used to excite the structure. The refractive index of the SiO_2_ layer is chosen as 1.45 and the permittivity of gold is obtained by fitting the experimental data from the literature^39^. Figure 1c shows the calculated scattering spectra of the toroidal structure under radially polarized light (See Methods). A resonance peak appears at 785 nm in the scattering spectrum with a full-width at half-maximum (FWHM) of 120 nm. The optical response of the designed structure is also investigated under the excitation of linearly polarized light, where a resonance peak is observed at 750 nm with a FWHM of 150 nm (Figure S1a, Supporting Information). The different FWHMs under the two incident lights indicate the excitation of a lower loss mode under radially polarized light.

To understand the inside physics of the difference, the magnetic field distributions at the resonance peaks under the two optical excitations are calculated. It is known that, a gold disk and a bottom layer separated by a dielectric spacer can be regarded as a magnetic resonator (MR), in which antiparallel currents are excited in two gold layers, resulting in a strong magnetic dipole moment^40^,^41^. For the radially polarized light, a toroidal response characterized by a closed loops of the magnetic field is clearly observed (Fig. 1d). While for the linearly polarized light, the vectors of the magnetic field are split in two bundles at the left and right halves of the structure, leading to a net magnetic dipole along the *y* direction (Figure S1b, Supporting Information). The different FWHM values under the two optical excitations can be easily understood since the toroidal dipolar response has a higher quality factor as compared with that for a magnetic dipole mode^17^.

**Figure 2:**
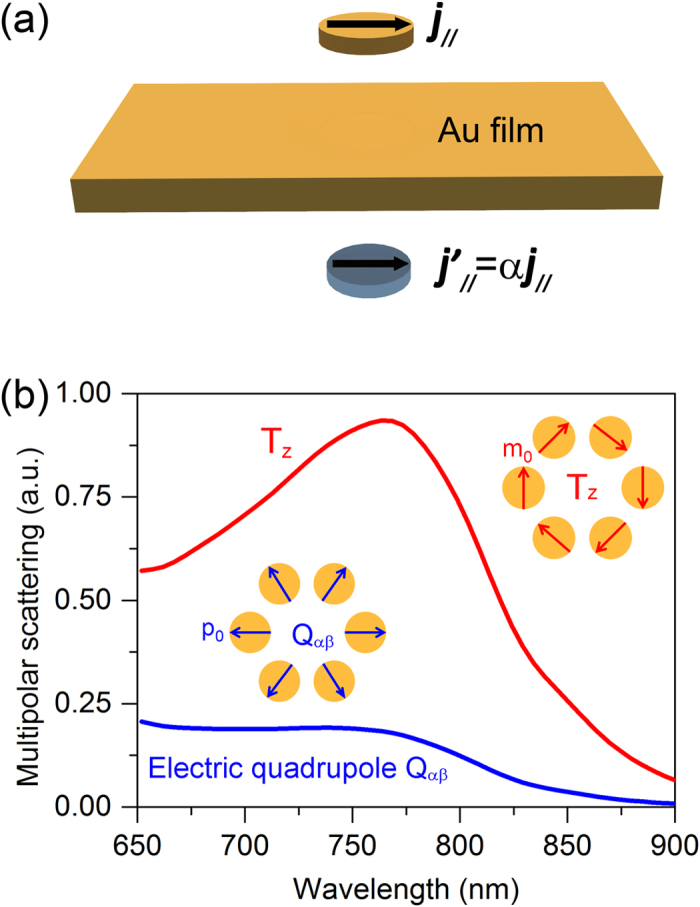
Calculations of the scattering power from multipoles. (**a**) Schematic of the mirror image***j***’_//_ for an in-plane current dipole source ***j***_//_ placed above a lossy metallic ground. (**b**) The arrangements of the residual electric dipoles and induced magnetic dipoles of the six MRs. (**c**) The calculated scattering powers for various multipole moments calculated from the currents in the upper gold hexamer and its image.

Although the toroidal dipolar response is observed, it should be made clear that if there are other multipoles, other than the toroidal moment, contributing significantly to the scattering spectrum. The conventional way is to calculate and compare the scattering powers of various multipolar moments by using the induced current in the metal. However, this method is difficult to deal with structure involving a metallic substrate. When a scatterer is placed above a planar metallic surface, the light is scattered into the out-of-plane propagating waves and surface plasmon-polariton (SPPs) waves propagating along the surface^42^,^43^. In this case, the currents in the metallic substrate include various components, such as the background field, the scatterer-induced field and the SPPs field, which cannot be used to calculate the multipolar moments. Nevertheless, for an in-plane current source ***j***_//_ placed above a lossy metallic ground (Fig. 2a), the field in the upper half-space can be considered as the superposition of contributions from the real source ***j***_//_ and its mirror image ***j’***_//_ = α***j***_//_. The correction factor *α* is given by^44^


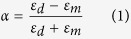


where *ε*_*d*(*m*)_ is the relative permittivity of the dielectric in the upper half space (the metallic ground). For a perfect electrical conductor (PEC) ground with *ε*_*m*_ → + ∞*i*, the correction factor is*α* = −1. Since the mirror image ***j***_//_ cannot totally offset the real source current ***j***_//_ in the optical frequency, each MR possess a residual electric dipole *p*_*0*_ and a magnetic dipole *m*_*0*_.

Under the illumination of radially polarized light, the six magnetic (electric) dipoles are azimuthally (radially) oriented at the vertexes of a hexagon, resulting in non-zero multipolar moments of electric quadrupole and toroidal dipole, as shown in inset of Fig. 2b. Due to the symmetry of the six electric and magnetic dipoles, the multipoles of the net in-plane electric dipoles***p***_//_, magnetic dipoles and magnetic quadruples are all zero. The scattering powers of the multipolar moments are then calculated from the induced currents in the upper gold hexamer and its mirror image, as shown in Fig. 2b. As the figure shows, the *z* component of the toroidal moment ***T***_***z***_ gives the dominant contribution in the entire considered frequency range and reaches its maximum at the resonance wavelength, demonstrating that the scattering power is mainly arisen from the toroidal dipole.

**Figure 3:**
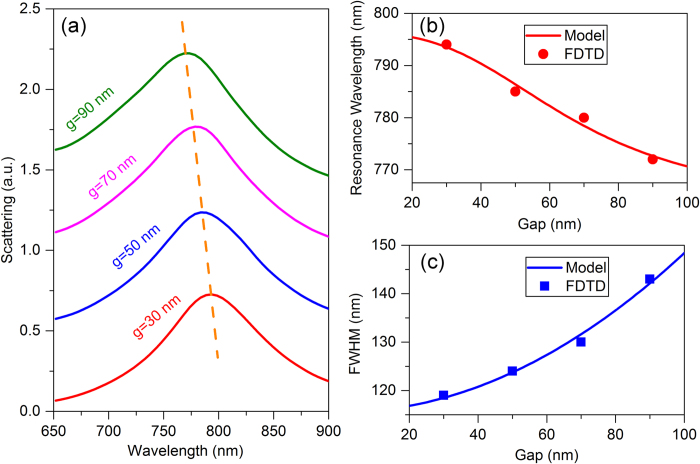
Optical responses of the toroidal structure with different gap size. (**a**) The FDTD calculated scattering spectra for the gold hexamer with the gap size ranging from 30 nm to 90 nm under radially polarized light. The offset between curves in (**a**) is 0.5. Dashed lines are drawn to guide the eye of the resonance peaks. The resonance wavelength (**b**) and the FWHM (**c**) of the toroidal mode dependence on the gap size. The solid circles and squares are the FDTD simulated data and the solid lines are the fittings of the model.

In Fig. 3a, we present the evolution of the calculated scattering spectra with the gap size *g* (defined in Fig. 1a) varying from 30 nm to 90 nm under radially polarized light. The resonance wavelength of the toroidal dipole mode is extracted and plotted in Fig. 3b (red filled circles), which shows a blue-shift with the increasing of the gap *g*. To understand the coupling of the six MRs, A Lagrangian hybridization model is adopted. The Lagrangian of the coupled system can be written as^45^


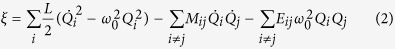


where *L* and *ω*_0_ are the inductance and resonance frequency of a single MR, *Q*_*i*_(*Q*_*i*_) represents the charge (current) on the *i*th MR, *M*_*ij*_ and *E*_*ij*_ are the mutual inductances for the magnetic and electric interactions between the *i*th and *j*th MRs, respectively. Based on the orientations of the six electric and magnetic dipoles (inset of Fig. 2b), the resonance frequency of the coupled system can be obtained as (see details in the Supporting Information)


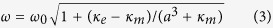


where *a* is the size length of the hexagon (*a* = *d* + *g*, *d* is the diameter of the gold disk, 120 nm), *κ*_*m*_ and 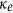 represent the coupling coefficients of the overall magnetic and electric dipole-dipole interactions, respectively. It is obvious that the interaction between magnetic dipoles is attractive while the interaction between electric dipoles is repulsive. The blueshift with the increasing of the gap indicates that the magnetic dipole-dipole coupling strength *κ*_*m*_ exceeds the electric dipole-dipole coupling strength *κ*_*e*_, showing that the overall coupling is attractive. Thus with increasing of the gap, the coupling is less attractive and the resonance wavelength blueshifts.

By fitting the data of the resonance wavelength (red solid line in Fig. 3b), the coupling strengths are easily to be calculated out as *κ*_*m*_ = 7.5 × 10^6^ *nm*^3^ and *κ*_*e*_ = 6.3 × 10^6^ *nm*^3^, which indeed shows *κ*_*m>*_*κ*_*e*_. To further demonstrate the correctness of this model, we consider a gold hexamer structure without the gold mirror, which is illuminated by radially polarized light. Since only the electric dipole-dipole interactions are involved in the system, the resonance wavelength is expected to exhibit a red-shift with increasing the gap, which is verified by the FDTD simulation (see details in Supporting Information).

The FWHM of the toroidal dipolar resonance as a function of the gap *g* is shown in Fig. 3c (blue filled squares). The FWHM Δ*f* is associated with the *Q* factor and is defined as Δ*f* = *f*/*Q* = *f*Δ*E*/2*πE*^46^, where *f* is the frequency, *E* is the energy stored in the structure and Δ*E* is the dissipated energy. The dissipated energy Δ*E* is the sum of the Ohmic losses Δ*E*_*m*_ in the metal and radiation losses Δ*E*_*r*_. For simplicity, we consider the Ohmic losses Δ*E*_*m*_ and the stored energy *E* to be independent on the gap *g*. From the arrangement of the electric and magnetic dipoles of MRs, we can directly calculate the radiation losses as (see details in Supporting Information)


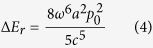


for the electric quadruple and


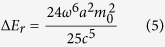


for the *z* component of the toroidal dipole ***T***_***z***_. Thus the FWHM Δ*λ* can be written as


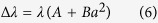


where *A* and *B* are two parameters, *λ* is the resonance wavelength. By fitting the FDTD data with the analytical prediction of Eq. 6, we can estimate the parameters as *A* = 0.112 and *B* = 1.6 × 10^−6^ *nm*^−2^. The fitting curve in Fig. 3c (blue solid line) agrees well with the results of FDTD simulation (blue solid squares).

**Figure 4:**
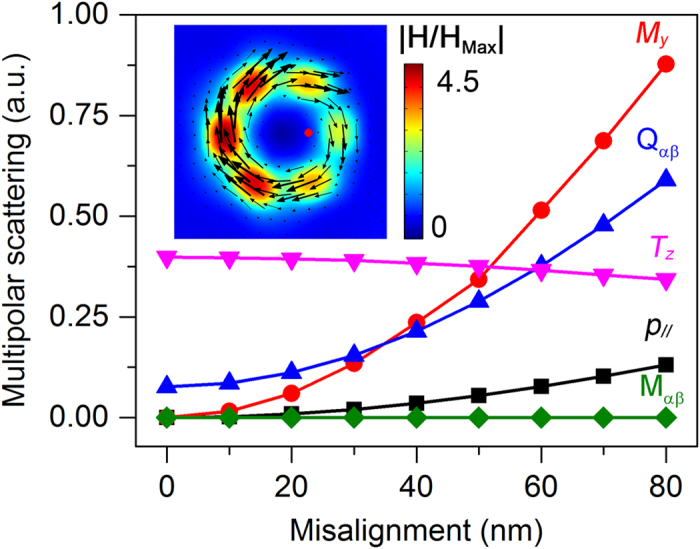
The misalignment tolerance property of the toroidal structure. The scattering powers from the multipoles as a function of the misalignment between the structure center and the incident light focus. The inset figure shows the magnetic field distributions when the beam center is misaligned of 50 nm (red solid circle). The black arrows represent the vectors of the magnetic fields.

In the above analysis, we consider the beam center to overlap with that of the hexamer. However, at nanometer scale such alignment is difficult and some deviations may occur in practice. In order to investigate the optical response of the designed toroidal structure under such deviations, the scattering power from the multipoles as a function of the misalignment between the structure center and the incident light focus at the toroidal dipolar resonance is shown in Fig. 4. The contribution from the magnetic quadrupole M_αβ_is almost zero for different misalignments. For ***T***_***z***_, its scattering power almost maintains constant with the increasing of the misalignment. When the beam and the hexamer are not perfectly aligned, the scattering powers from the *y* component of the magnetic dipole ***M***_***y***_ and the in-plane of the electric dipole ***p***_**//**_ emerge and increase with the increasing of the deviation, as well as the electric quadrupole Q_αβ_. The sharpest increase is observed for ***M***_***y***_, mainly as a consequence of the asymmetry breaking of the magnetic dipoles. Although the ratio of ***T***_***z***_ in the total scattering power decreases as the misalignment is increased, it still gives the dominant contribution when the deviation is less than 50 nm, showing a robust, misalignment tolerant properties of the designed structure. The inset figure shows the magnetic field distributions when the beam center is misaligned of 50 nm, where a clear toroidal dipolar response can still be observed.

## Discussion

We theoretically studied a novel MIM structure that exhibits resonant toroidal dipolar response in the optical frequency. Our configuration is based on the magnetic resonance of a gold disk placed on a gold mirror separated by a dielectric layer. By illuminating a radially polarized light, the designed structure is capable of suppressing the components of electric and magnetic dipole moments due to the symmetries of the incident light and structure. We show that the toroidal moment formed by a closed loop of the magnetic dipoles gives the dominant contribution in the scattering spectrum, which is much higher than that of the electric quadruple arising from the net electric dipoles due to the non-perfect mirror image of the gold disk. By investigating the frequency shift of the toroidal mode dependence on the gap of the gold hexamer, we further demonstrate that the magnetic dipole-dipole coupling exceeds the electric dipole-dipole coupling. Our study opens the way for the realization of unique resonance modes, combining complex plasmonic structures with the remarkable properties of cylindrical vector beam.

## Methods

### Simulation

The numerical computations were carried out by the software FDTD method. The radially polarized light has a doughnut-shape field distribution of *E*_0_·*r*·exp(−*r*^2^/*σ*^2^), where *σ* is the beam waist and is assumed to be 250 nm in the calculations. For the calculation of the scatter spectrum, we first computed a background fields from the radially polarized light incident on the substrate, and then calculated the total fields with the Au nanostructures present. The scattered fields were defined as the differences between the total fields and the background fields.

## Additional Information

**How to cite this article**: Bao, Y. *et al.* Plasmonic Toroidal Dipolar Response under Radially Polarized Excitation. *Sci. Rep.* **5**, 11793; doi: 10.1038/srep11793 (2015).

## Supplementary Material

Supporting Information
