# Supplementary material for: Plasmonic Toroidal Dipolar Response under Radially Polarized Excitation
Source: Sci Rep. 2015 Jun 26;5:11793. doi: 10.1038/srep11793 (PMC4481838; doi:10.1038/srep11793)
Supplement: Supporting Information [file srep11793-s1.doc]

Supplementary information

Plasmonic Toroidal Dipolar Response under Radially Polarized Excitation

Yanjun Bao, ^1^ Xing Zhu, ^1^ and Zheyu Fang ^1, 2^,*

^1^School of Physics, State Key Lab for Mesoscopic Physics, Peking University, Beijing 100871, China

^2^Collaborative Innovation Center of Quantum Matter, Beijing 100871, China

[*zhyfang@pku.edu.cn](mailto:*zhyfang@pku.edu.cn)

1. **The optical response of the toroidal structure under linearly polarized light**

**
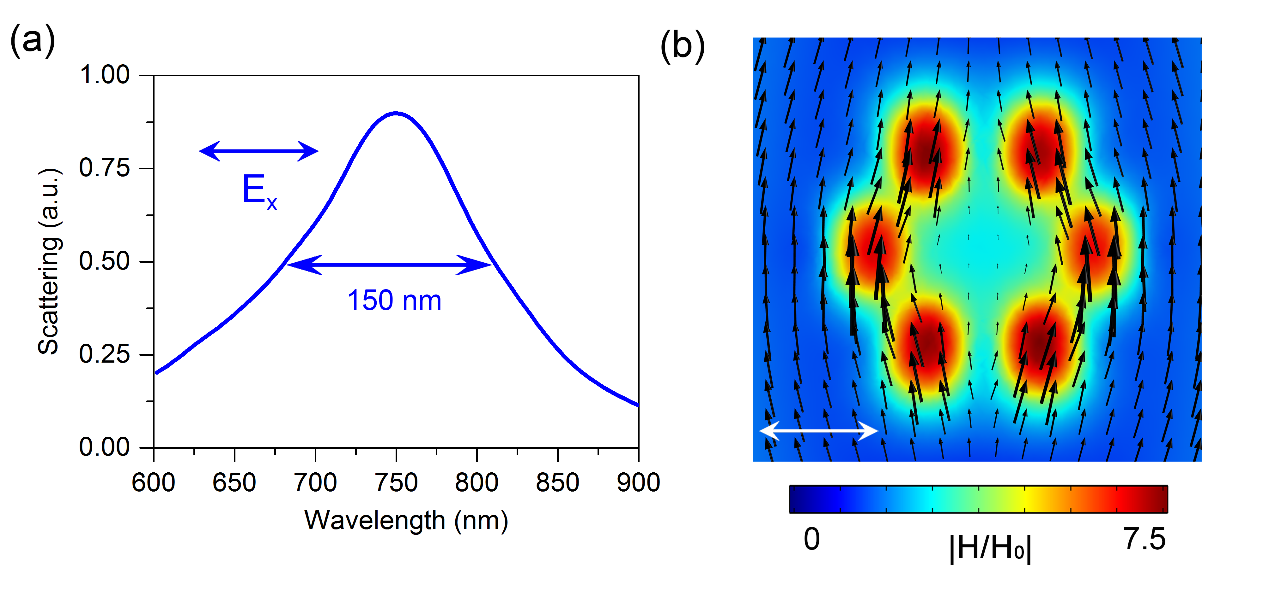
**

Figure S1 (a) The FDTD calculated scattering spectra of the toroidal structure under linearly polarized light. (b) The simulated magnetic field at the center of the dielectric layer at the resonance peak. The black arrows represent the vectors of the magnetic fields.

1. **The Lagrangian hybridization model of the toroidal metamaterial**

The Lagrangian of the coupled system can be written as

(S1)

where $L$ and $\omega_{0}$ are the inductance and resonance frequency of a single magnetic resonator (MR), represents the charge (current) on the *i*th MR, $M_{ij}$and$E_{ij}$are the mutual inductances for the magnetic and electric interactions between the *i*th and *j*th MRs, respectively.

Substituting the above equation to the Euler-Lagrangian equations,, and adopt the root form of $Q_{i}=A_{i}e^{i\omega t}$, we get

$\omega_{i}=\omega_{0}\sqrt{\frac{L+\sum_{j\neq i} E_{ij}}{(L-\sum_{j\neq i} M_{ij})}}$ (S2)


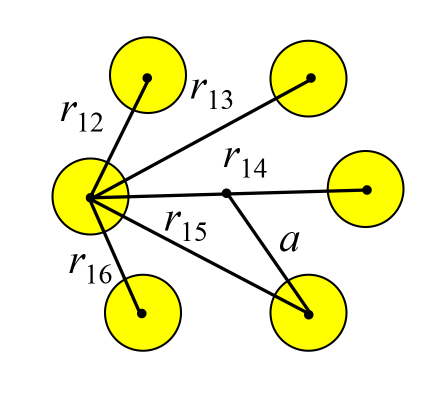


Figure S2. The geometry of the six MRs and the representations of the distances between two MRs.

The coupling coefficients of magnetic and electric dipole-dipole $M_{ij}$ and $E_{ij}$ can be calculated by using the energy of the dipole-dipole interaction, which has the form of

$E_{dipole}=\frac{\mathbf{d}_{i}\cdot\mathbf{d}_{j}}{r^{3}}-3\frac{(\mathbf{d}_{i}\cdot\mathbf{r})(\mathbf{d}_{j}\cdot\mathbf{r})}{r^{5}}$ (S3)

where $\mathbf{d}_{i}$ represents either the magnetic (, *S* is the effective area of the current loop of the MR) or the electric ($\mathbf{p}_{i}=Q_{i}l$, *l* is the effective distance between positive and negative charges of the MR) dipole of the *i*th MR, and *r* is the distance between the *i*th and *j*th MRs. Without losing generality, we take the *1*th MR as an example, the distances between it and the other five MRs are respectively (Figure S2),

$r_{12}=r_{16}=a;$ $r_{13}=r_{15}=\sqrt{3}a;$ $r_{14}=2a;$ (S4)

Based on the orientation of the magnetic and electric dipoles in inset of Figure 2b, the coupling coefficients can be calculated as:

$M_{12}=M_{16}=-\frac{7S^{2}}{4c^{2}r_{12}^{3}},$ $M_{13}=M_{15}=-\frac{5S^{2}}{4c^{2}r_{13}^{3}},$ $M_{14}=-\frac{S^{2}}{c^{2}r_{14}^{3}},$ (S5a)

$E_{12}=E_{16}=\frac{5l^{2}}{4\omega_{0}^{2}r_{12}^{3}},$ $E_{13}=E_{15}=\frac{7l^{2}}{4\omega_{0}^{2}r_{13}^{3}},$ $E_{14}=\frac{2l^{2}}{\omega_{0}^{2}r_{14}^{3}}.$ (S5b)

Summing the five terms, we get

$\sum_{j\neq i} M_{1j}\approx\frac{-4.106S^{2}}{c^{2}a^{3}}=\frac{-\kappa_{m}L}{a^{3}}$ (S6a)

$\sum_{j\neq i} E_{1j}\approx\frac{3.147l^{2}}{\omega_{0}^{2}a^{3}}=\frac{\kappa_{e}L}{a^{3}}$ (S6b)

where $\kappa_{m}=4.106\frac{S^{2}}{c^{2}L}$ and $\kappa_{e}=3.147\frac{l^{2}}{\omega_{0}^{2}L}$ represent the coupling coefficients of the magnetic and electric dipole-dipole, respectively. The negative sign of Eq. S6a indicates that magnetic dipole-dipole interaction is attractive, while the positive sign of Eq. S6b indicates that electric dipole-dipole interaction is repulsive. The resonance frequency of the coupled system then can be obtained as

$\omega=\omega_{1}=\omega_{0}\sqrt{1+(\kappa_{e}-\kappa_{m})/(a^{3}+\kappa_{m})}$ (S7)

1. **The scatter spectra of a gold hexamer with different gap size**

The gold hexamer placed on the SiO_2_ substrate is illuminated by a radial polarized light, in which only the electric dipole-dipole interactions exist. Thus the resonance frequency can be obtained as:

$\omega=\omega_{1}=\omega_{0}\sqrt{1+\kappa_{e}/a^{3}}$ (S8)

Figure S3b shows a redshift with the increasing of the gap size varying from 30 nm to 90 nm, which has a good agreement with the prediction of Eq. S8.


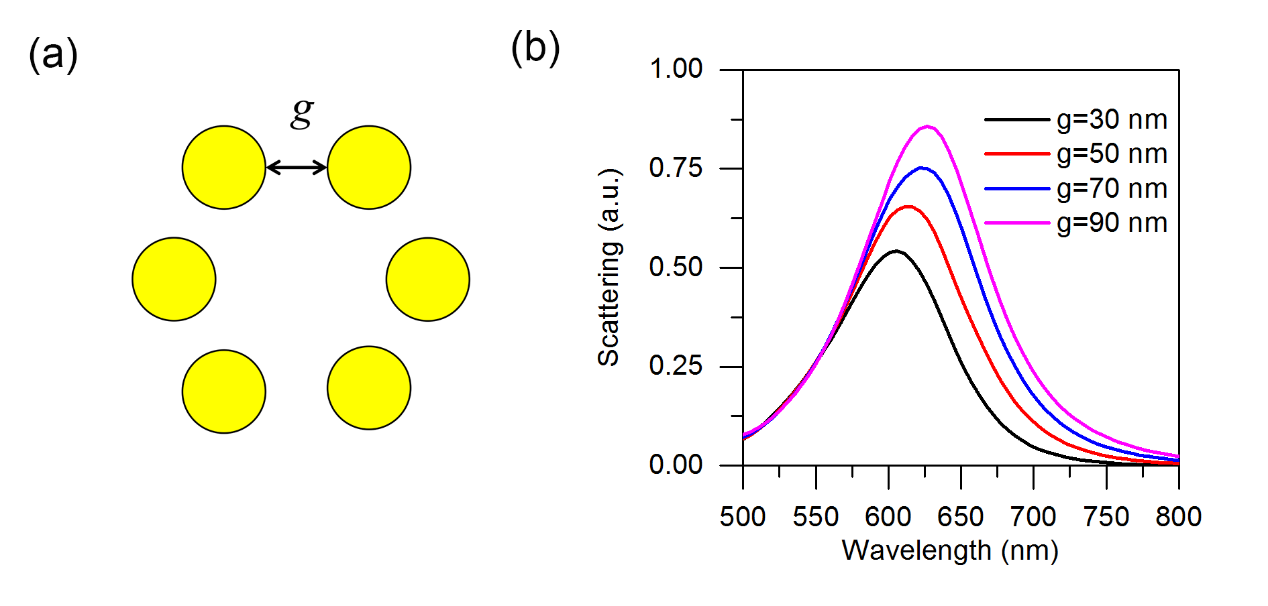


Figure S3. (a) The geometry of a gold hexamer with gap size *g* illuminated by radial polarized light. The diameter of the disk is 120nm. (b) The scatter intensity with the gap varying from 30 nm to 90 nm.

**4. The radiation powers of the electric quadrupole and toroidal dipole**

The electric quadrupole can be rewritten in the electric dipoles as

$Q_{\alpha\beta}=\frac{1}{2}\sum_{i} [r_{i\alpha}p_{i\beta}+r_{i\beta}p_{i\alpha}-\frac{2}{3}(\mathbf{r}_{i}\cdot\mathbf{p}_{i})\delta_{\alpha\beta}]$ (S9)

For the six electric dipoles shown in inset of Figure 2b, the non-zero components of the electric quadrupole are

$Q_{11}=Q_{22}=2ap_{0}$, (S10)

Thus the radiation power of the electric quadrupole is

$\Delta E_{r}=\frac{\omega^{6}}{5c^{5}}\sum\left|Q_{\alpha\beta}\right|^{2}=\frac{8\omega^{6}a^{2}p_{0}^{2}}{5c^{5}}$ (S11)

The *z* component of the toroidal dipole formed from the six magnetic dipoles shown in inset of Figure 2b can be rewritten as

$T_{z}=\frac{1}{5}\sum_{i} (\mathbf{r}_{i}\times\mathbf{m}_{\mathbf{i}})=\frac{6}{5}am_{0}$ (S12)

Then the radiation power of toroidal dipole is

$\Delta E_{r}=\frac{2\omega^{6}}{3c^{5}}\left|\mathbf{T}_{z}\right|^{2}=\frac{24\omega^{6}a^{2}m_{0}^{2}}{25c^{5}}$ (S13)
